# Cryopreserved rabbit amniotic membrane alleviated inflammatory response and fibrosis following experimental strabismus surgery in rabbits

**DOI:** 10.1371/journal.pone.0187058

**Published:** 2017-10-24

**Authors:** Bo Young Chun, Soolienah Rhiu

**Affiliations:** 1 Department of Ophthalmology, Kyungpook National University Hospital, Kyungpook National University School of Medicine, Daegu, Korea; 2 Brain science & Engineering Institute, Kyungpook National University School of Medicine, Daegu, Korea; 3 Department of Ophthalmology, Dongtan Sacred Heart Hospital, Hallym University College of Medicine, Hwaswong, Korea; Ocular Surface Center, UNITED STATES

## Abstract

We evaluate the ability of cryopreserved rabbit amniotic membrane (AM) transplantation to reduce postoperative inflammation and the extent of fibrosis following experimental strabismus surgery. Ten white rabbits underwent bilateral superior rectus (SR) muscle resection. In the left eye, the resected SR muscle was wrapped with cryopreserved rabbit AM. The right eye underwent SR resection only and served as a control. The eyes were enucleated 4 weeks after strabismus surgery. The degree of postoperative inflammatory infiltration, the extent of fibrosis, and profile of the relative expression of inflammatory mediators in the SR muscle were evaluated and compared between the two groups by histological analysis and real-time polymerase chain reaction (PCR). There were statistically meaningful differences in the degree of postoperative inflammatory infiltration and extent of fibrosis between the eyes treated with cryopreserved rabbit AM after SR resection and those underwent SR resection only. A significant decrease in the expression of inflammatory cytokines [interleukin (IL)-12a, IL-12b, IL-17f, and tumor necrosis factor- alpha (TNF-α)], and a markedly increased expression of anti-inflammatory cytokines (transforming growth factor-beta-1(TGFβ-1) and IL-10) were observed in the eyes treated with cryopreserved rabbit AM. In this study, we demonstrate that cryopreserved rabbit AM is effective in reducing postoperative inflammation and extent of fibrosis in a rabbit model of strabismus surgery. Our results imply that cryopreserved AM allograft has anti-inflammatory and anti-scarring properties that can prevent postoperative adhesions following strabismus surgery.

## Introduction

One of the main complications following strabismus surgery is postoperative adhesions, which can affect the outcome of surgery by restricting motility of the eyeball [1, 2]. To prevent further fibrovascular proliferation and fibrosis following strabismus surgery, numerous attempts have been introduced to suppress postoperative inflammatory infiltration, including mechanical barrier materials and application of steroids, anti-proliferative chemical agents, or viscoelastic substances [3–9]. However, none of these techniques could be applied in clinical practice because of complications or unpredictable postoperative results.

The amniotic membrane (AM) is a thin and transparent film-like tissue of the fetal side of the placenta, and consists of an epithelium, a basement membrane, and an adjacent avascular stromal matrix [3, 10–12]. Lately, the use of AM has been increasing in various fields of ophthalmology, including surgical treatment of pterygium, ocular surface reconstruction and glaucoma filtration surgery such as trabeculectomy [3, 13–15]. Application of the AM reduces inflammation, scarring, and neovascularization in several ocular surface disorders by decreasing the mixed lymphocyte reaction, suppression of the interleukin (IL)-1-mediated inflammatory cascade, and delayed hypersensitivity reaction [10–12, 16].

Thus, several researches have been performed on the role of the AM as a biological barrier in strabismus surgery by reducing postoperative inflammation and scarring. However, our previous research demonstrated that lyophilized human AM was not efficient in alleviating postoperative inflammation and fibrosis following strabismus surgery in rabbits, and those data corresponded with the results reported by Kassem et al. [17,18] We speculated that these results might be due to the limited benefit of lyophilized human AM [19]. Kassem et al. [20] reported that cryopreserved human AM was effective in reducing postoperative adhesions in rabbits. However, differences were not significant in postoperative conjunctival inflammation and muscle fibrosis. Until now, there is no experimental data evaluating the anti-inflammatory and anti-fibrosis effects of cryopreserved rabbit AM allografts in an experimental model of strabismus surgery to eliminate possible xenogenic effect.

Therefore, we evaluated the ability of cryopreserved rabbit AM to reduce the postoperative inflammation and fibrosis following experimental strabismus surgery in rabbits in this study.

## Materials and methods

### Animals

Ten New Zealand white rabbits weighing 2.5–3 kg were used in this experimental study. Animals used in this experiment were acquired and cared for according to the procedures approved by the Institutional Animal Care Committee of Kyungpook National University, and the animal care guidelines of the National Institute of Health (NIH; Bethesda, MD, USA), and in accordance with the ARVO Statement for the Use of Animals in Ophthalmic and Vision Research.

### Preparation of cryopreserved rabbit AM

Cryopreserved rabbit AM was prepared according to the previously reported method. [21] The placentas were obtained by veterinary surgeons following elective caesarean section in two full-term pregnant rabbits. The AM was separated carefully from the chorion under aseptic conditions. The AM was rinsed out several times with sterile phosphate-buffered saline (PBS) (Invitrogen, Grand Island, NY, USA) with a mixture of streptomycin 50 μg/ml, penicillin 50 μg/ml, amphotericin B 2.5 μg/ml, and neomycin 100 μg/ml to remove blood clots. The AM was placed epithelial side up (a shiny and smooth surface) on nitrocellulose paper prior to cutting the sample into appropriately sized pieces. The pieces of AM were stored in cryomedium consisting of 500 ml of Dulbecco’s modified Eagles medium (DMEM) (Invitrogen) and 500 mg of glycerol at -80°C for 1 month prior to use.

### Surgery

The rabbits were anesthetized by an intramuscular injection with a mixture of ketamine hydrochloride 20 mg/mL (Ketalar^®^, Parke Davis, New York City, NY, USA) at 2.5 mg/kg and an aqueous solution of 2% xylazine 7 mg/mL (Rompum^®^, Bayer, Leverkusen, Germany) at 5 mg/kg. The level of anesthesia was monitored by the toe withdrawal reflex and blink reflex, and additional doses were added if required. Superior rectus (SR) muscle resection was performed in both eyes in all rabbits. All surgeries were performed by the same surgeon (BYC). A povidone-iodine solution was applied on the eyelids for preoperative antisepsis. The SR muscle was isolated on a muscle hook via a fornix-based limbal incision. The intermuscular septums and superior oblique muscle were then dissected carefully from the SR muscle with Westcott scissors. A 6–0 absorbable polyglactin (Vicryl^®^) was sutured on the SR muscle 1 mm behind the resection point, and the SR muscle resection of 4 mm was performed. To aggravate the inflammatory response following this experimental strabismus surgery, cauterization of the underlying sclera (1 × 0.5 cm^2^, 10 times) was done prior to suturing of the resected SR muscle to its original insertion [17, 22]. The reason for this additional cauterization is that the average degree of inflammation following strabismus surgery is unknown in rabbits, although De Carvalho et al. [7] demonstrated that postoperative inflammation following uncomplicated strabismus surgery was minimal in this species. Hence, we augmented the postoperative inflammatory response by additional procedure to more easily demonstrate the anti-inflammatory property of the cryopreserved AM. The right eye of each rabbit underwent a 4-mm SR resection and additional cauterization of the underlying sclera without using AM (group C). In the left eye, the resected SR muscle was wrapped with a layer of cryopreserved rabbit AM prior to the reattachment of SR muscle to its original insertion (group AM). The relatively coarse and adhesive surface (stromal side) of the AM was placed towards the muscle. Due to the fact that fibrosis proliferation was expected to originate from fibroblasts of the subconjunctiva [1, 9], the conjunctiva of all eyes were then carefully repositioned and closed with two 6–0 sutures. During surgery, the surgeon chose which eye to operate on first at random. After surgery, the condition of the rabbits was monitored, and an antibiotic ointment (Terramycin^®^) was applied to the eyes of the rabbits every day. None of the rabbits showed severe signs of illness following surgery.

### Enucleation and histopathologic examination

**Fig 1:**
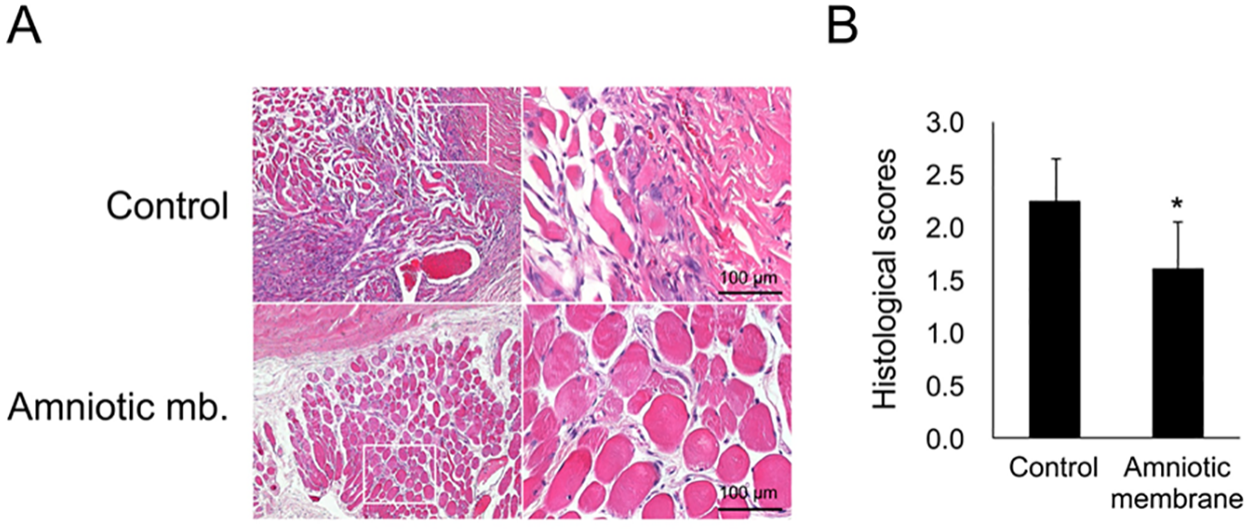
Comparison of the severity of postoperative inflammation after experimental strabismus surgery between group C (control group) and group AM (Amniotic membrane). (A) *Left*, representative photographs of the superior rectus muscle and surrounding connective tissue with H&E stain and 100x and 400x magnification. (B) *Right*, There was a statistically significant difference in mean inflammatory score between the control eyes (2.25 ± 0.41) and AM eyes (1.61 ± 0.45; *p* = 0.048).

**Fig 2:**
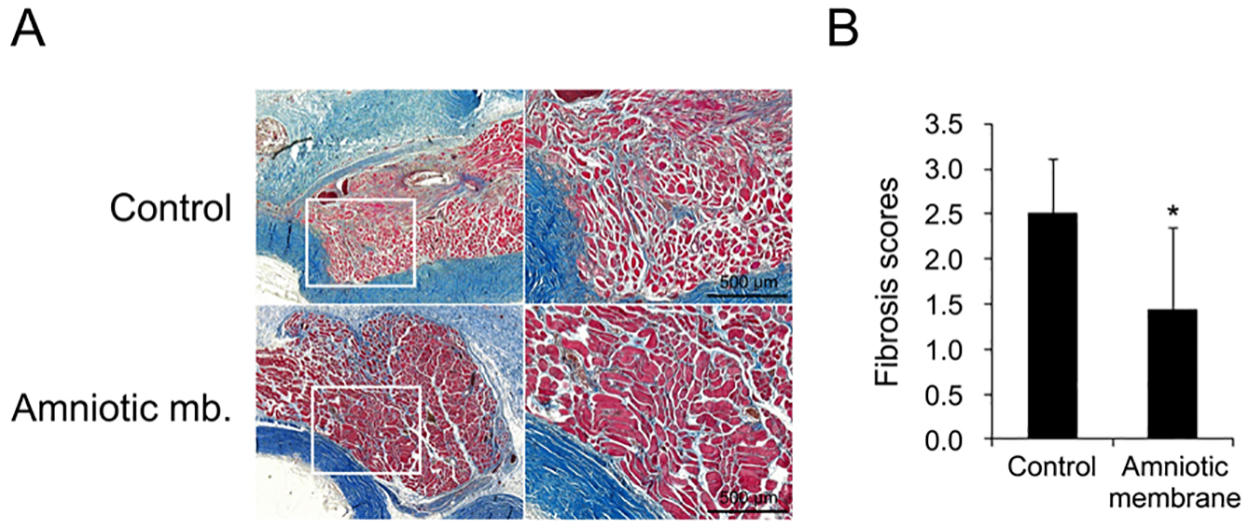
Comparison of the severity of postoperative fibrosis after strabismus surgery between group C and group AM. (A) Left, representative photographs of the superior rectus muscle and surrounding connective tissue with Masson Trichrome stain and 80x and 200x magnification. (B) *Right*, there was a statistically significant difference in mean fibrosis score between the control eyes (2.50 ± 0.61) and the AM eyes (1.43 ± 0.93; *p* = 0.015).

Four weeks following the surgery, all rabbits were anesthetized again. The location of the SR was marked at the limbus with marking pen, and then the eyes were enucleated with caution to avoid damage to the new insertion site of the resected SR muscle. An incision parallel to the limbus was made at the superior conjunctiva 12 mm posterior to the limbus. Next, the incision was vertically extended to the 3 and 9 o’clock positions and the SR muscle was excised including the surrounding conjunctiva and Tenon’s capsule. The rabbits were euthanized with intravenous KCL (1-2meq/kg) injection under anesthetic state. For histopathologic examination, half of the excised tissue samples were fixed in 10% buffered formaldehyde and embedded in paraffin. Six coronal sections for each eye were made parallel to the SR muscle reinsertion. The tissues were processed for histologic examination by staining with Masson’s trichrome and hematoxylin-eosin to evaluate the severity of postoperative inflammatory infiltration and the extent of fibrosis. The degree of inflammatory infiltration was graded by semi-quantitative analysis with a light microscope, by a pathologist who was blinded to the experiment design of this study (Figs 1 and 2) [7, 17, 23]. The subsequent grading system reported previously was used in this study [7, 17, 23]: Grade 0 (absent inflammatory infiltration}; Grade 1 [mild inflammatory infiltration (presence of lymphocytes)]; Grade 2 [moderate inflammatory infiltration (presence of lymphocytes, macrophages, and plasmocytes)]; and Grade 3 [intense inflammatory infiltration (presence of lymphocytes, macrophages, plasmocytes and neutrophils)].

After scoring the degree of inflammation in six sections per eye, the average value was calculated to compare the mean value of grades of inflammatory infiltration between the two groups.

The collagen deposition between the SR muscle and the surrounding tissue was defined as fibrosis, which was stained blue with Masson’s trichrome. The subsequent grading system was used in this study: Grade 0 (no observable fibrosis); Grade 1 (presence of fibrous tissue between the sclera and SR muscle); Grade 2 (presence of fibrous tissue between the sclera and SR muscle + presence of fibrous septa between SR muscle fibers); and Grade 3 (presence of fibrous tissue between the sclera and SR muscle + presence of fibrous septa between SR muscle fibers + fibrous tissue expansion through Tenon’s capsule and the subconjunctival space).

After scoring the extent of fibrosis in six sections per eye, the average value was calculated to compare the mean value of grades of fibrosis between the two groups.

### Real-time polymerase chain reaction

According to the manufacturer's instructions, total RNA was extracted from the other half of the resected SR muscle, using TRIzol reagent (Invitrogen). Total RNA (0.5 μg) was reverse-transcribed into cDNA, with Superscript II (Invitrogen) and oligo (dT) primers. Polymerase chain reaction (PCR) amplification was performed with a DNA Engine Tetrad Peltier Thermal Cycler (MJ Research, Waltham, MA, USA) at a temperature of 55–60°C for 20–30 cycles, with specific primer sets. For the analysis of the PCR products, 10 μl of each was electrophoresed on 1% agarose gel and detected under ultraviolet (UV) light. GAPDH was used as the internal control. Real-time PCR was done with the One Step SYBR^®^ PrimeScript^™^ RT-PCR kit (Perfect Real-Time; Takara Bio Inc., Tokyo, Japan), and detection was done with the ABI Prism^®^ 7500 Sequence Detection System (Applied Biosystems, Foster City, CA, USA).

### Statistical analysis

Mean scores of the degree of inflammatory infiltration and the extent of fibrosis were analyzed by the Mann-Whitney test. All other values are expressed as the means ± standard deviation. The Student’s *t*-test was done to determine the statistical significance of differences in relative expression of inflammatory mediators between group C and group AM. The statistical analyses were performed using SPSS version 12.0 software (SPSS, Inc., Chicago, IL, USA). In all tests, statistical significance was accepted for *p*-values < 0.05.

## Results

### Comparison of histologic examinations between groups

Fig 1 shows a comparison of the degree of postoperative inflammatory infiltration following experimental strabismus surgery between group C and group AM. The degree of inflammatory infiltration was scored in each eye. There was a statistically meaningful difference in mean inflammatory score between the control eyes (2.25 ± 0.41) and AM eyes (1.61 ± 0.45; *p* = 0.048).

Fig 2 shows a comparison of the degree of postoperative fibrosis following strabismus surgery between group C and group AM. The degree of fibrosis was scored in each eye. There was a statistically significant difference in mean fibrosis score between the control eyes (2.50 ± 0.61) and the AM eyes (1.43 ± 0.93; *p* = 0.015). In group AM, the AM remained intact in eight eyes, as revealed by histological examination after 4 weeks.

### Relative expression of inflammatory and anti-inflammatory cytokines

**Fig 3:**
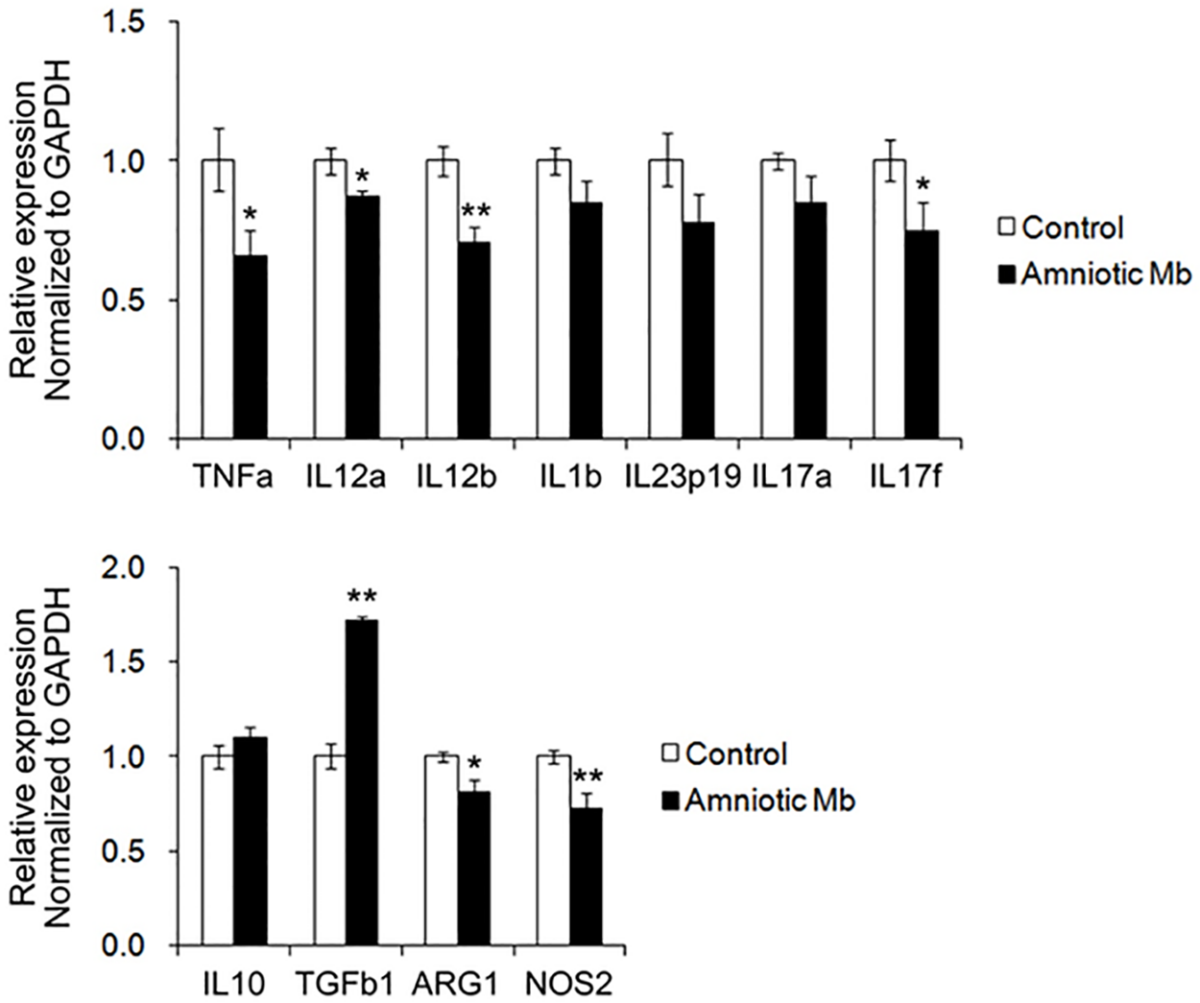
Relative expression of inflammatory and anti-inflammatory cytokines. Real-time PCR analysis was performed to assess the relative expression of inflammatory (TNF-α, IL-12a, IL-12b, IL-17f, IL-1b, IL-23p19, IL-17a), anti-inflammatory cytokines (IL-10, TGF- β1), and the oxidative cytokines (ARG1, NOS2) in the eyes from group C and AM. Data are mean ± SD. **P<0.01, *P<0.05, group C (control) versus group AM (amniotic membrane).

Real-time PCR analysis was done to assess the relative expression of inflammatory mediators in the eyes from group C and AM (Fig 3). Relative expression of all inflammatory cytokines measured in this study [tumor necrosis factor alpha (TNF-α), IL-12a, IL-12b, IL-17f, IL-1b, IL-23p19, IL-17a] was lower in group AM than those in group C; expression of TNF-α, IL-12a, and IL-17f (*p* < 0.05), as well as IL-12b (*p* < 0.01), was significantly decreased in the group AM eyes when compared with that in the group C eyes. Relative expression of the anti-inflammatory cytokines measured in this study [IL-10, transforming growth factor (TGF)-β1] was increased in the group AM eyes when compared with those in the group C eyes; TGF-β1 level was increased to a statistically significant level in the group AM eyes when compared with that in the group C eyes (p < 0.01). Relative expression of the oxidative cytokines measured in this study (ARG1 [*p* < 0.05], NOS2 [*p* < 0.01]) was also significantly decreased in the group AM eyes when compared with those in the group C eyes.

## Discussion

Considering that the wound healing process is defined as an inflammation in the acute phase and subsequent scarring in the chronic phase [24–26], an approach to decrease postoperative scarring and adhesions following strabismus surgery should focus on suppression of postoperative inflammation. The AM is an immunologically inactive tissue, and has been reported to reduce inflammation in a number of ocular surface diseases [3, 10, 11, 13–16, 21, 24, 26]. The AM has anti-inflammatory, anti-scarring, and anti-angiogenic effects by means of biochemically purified novel matrix components termed heavy chain (HC)-hyaluronan (HA)/pentraxin 3 (PTX3) complex, which may be the key factors responsible for the aforementioned AM’s characteristics [27].

In this study, we evaluated the effects of cryopreserved rabbit AM on inflammation and fibrosis following experimental strabismus surgery in rabbits. The eyes treated with cryopreserved rabbit AM demonstrated significantly less inflammatory infiltration than the control eyes. In addition, there was a statistically significant reduction in mean fibrosis scores in the AM-treated eyes when compared with that in the control eyes. The pivotal role of cryopreserved rabbit AM used in this experimental strabismus surgery as a promising biological barrier was corroborated by findings of significantly increased expression of anti-inflammatory cytokines such as TGF-β1 and IL-10 and decreased expression of inflammatory cytokines such as TNF-α, IL-12, and IL-17 in eyes treated with cryopreserved rabbit AM when compared with control eyes.

Our results correspond with those of previous studies demonstrating that the AM decreases postoperative adhesions following strabismus surgery. Sheha et al. [16] described a patient in whom wrapping the extraocular muscle (EOM) with a sheet of cryopreserved AM restored EOM motility following reoperation for strabismus. Kersey and Vivian [28] reported that concurrent application of mitomycin C and the AM decreased postoperative adhesions in two patients with complicated strabismus surgery, but with limited success. In addition, Kassem et al. [20] demonstrated that cryopreserved human AM was efficient in reducing postoperative EOM adhesions in rabbits; muscle adhesions were absent in eight eyes treated with cryopreserved human AM, and adhesions were detected in all ten control eyes. However, differences between the AM-treated eyes and control eyes were not significant in postoperative conjunctival inflammation and muscle fibrosis [20].

In contrast, there are several studies reporting that application of the AM did not reduce inflammation and fibrosis following strabismus surgery. Our previous experimental study [17] demonstrated that the use of lyophilized human AM was not efficient in alleviating postoperative inflammation and fibrosis following strabismus surgery in the rabbit eyes, and those data corresponded with the results reported by Kassem et al. [18]. They reported that adhesions were present in all eyes treated with lyophilized human AM when used to wrap the EOM in rabbits, limiting the benefit of lyophilized AM in strabismus surgery [18]. In addition, Kassem et al. used dried human AM during strabismus reoperation in a patient with residual esotropia, and reported an unfavorable outcome [29]. However, Mehendale and Dagi used a dried human AM in a patient with severe adhesions following orbital fractures and reported a favorable outcome [30].

There are two important features to note in this study. The first important aspect to note is that we used a rabbit-rabbit allograft transplantation model to eliminate any possibility of an undesirable xenograft rejection phenomenon between humans and rabbits. In our previous study, we encountered an unexpected nonsignificant tendency of increased inflammatory infiltration and fibrosis surrounding the applied dried human AM. Those findings may be a manifestation of unwanted xenograft rejection between humans and rabbits [31], although the dried human AM applied in the study did not carry any live cells [17]. In addition, Kassem et al. [20] reported that cryopreserved human AM was effective in preventing muscle adhesions, but was unsuccessful in preventing conjunctival inflammation and fibrosis of the rectus muscle. It was inferred that these findings might have been due to an attack on the AM by the host tissue, which was induced by the AM epithelium and probably related to the xenograft rejection [20, 31]. A significant decrease in postoperative inflammation and fibrosis was observed in this study after applying cryopreserved rabbit AM in experimental strabismus surgery, and it is inferred that those favorable results is due to using rabbit AM on rabbits to get rid of possible xenograft rejection.

Another important aspect to note in this study is that we used cryopreserved AM instead of lyophilized AM. It is well known that the AM contains a various cytokines and growth factors, which have favorable effects on inflammation, wound healing, and prevent neovascularization [10, 16, 17, 19]. The relative expression of all inflammatory cytokines measured in this study (TNF-α, IL-12a, IL-12b, IL-17f, IL-1b, IL-23, IL-17a) was decreased in group AM when compared with that in group C, and the relative expression of the anti-inflammatory cytokines measured in this study (IL-10, TGF-β1) was increased in the group AM when compared with that in group C. These results correspond with those in a study by Paolin et al. [32], who reported cytokine expression and ultrastructural alterations in human AM. During dry preparation of human AM, it may lose the expected anti-inflammatory and anti-fibrosis effects found in cryopreserved AM [17, 19]. There are reports that concentrations of IL-10 and TGF-β1 are not affected by the method applied to manufacture cryopreserved AM; these are crucial immunoregulatory cytokines with anti-inflammatory properties [19, 32–34]. TGF-β1 and IL-10 are suppressor cytokines produced by functional regulatory T-cells, and their main role is to generate immune tolerance by suppression of the immune reaction [33]. Therefore, we speculate that well-preserved anti-inflammatory cytokines in cryopreserved AM could suppress the inflammatory response induced by experimental strabismus surgery. In addition, cryopreserved AM has a layer of devitalized epithelium and better preservation of the basement membrane, whereas lyophilized AM is deprived of epithelial cells as a result of the manufacturing process [17, 19, 20, 32]. Epithelialized surfaces cannot adhere to other tissue, and adhesions detected in previous studies using lyophilized AM [17, 28] suggest that cryopreserved AM allografts may act as a better biological barrier than lyophilized AM for prevention of postoperative fibrosis and adhesions following strabismus surgery.

This study has a few critical limitations that mostly stem from its small number of animals and experimental use of rabbit AM. In addition, we did not perform immunohistochemistry study against smooth muscle actin, fibronectin, collagen I or collagen IV, which may help quantitative analysis of the degree of scar tissue or fibrosis. We could not discover novel mechanism but only described beneficial effects of cryopreserved rabbit AM used in the rabbits in this study. A further prospective clinical study in humans will be necessary to prove the role of cryopreserved human AM as a useful biological barrier against postoperative inflammation and fibrosis following strabismus surgery.

Although there were several studies using human AM in experimental strabismus surgery model, our study is the first experiment to evaluate the effect of cryopreserved “rabbit” AM against postoperative fibrosis by alleviating postoperative inflammatory infiltration in experimental strabismus surgery in rabbits. Cryopreserved AM seems to downregulate postoperative fibrosis by decreasing the expression of inflammatory cytokines and increasing the expression of anti-inflammatory cytokines. Thus, cryopreserved allograft AM holds promise as a potential therapeutic agent to provide a safe biological barrier in strabismus surgery.
